# TOM20-mediated transfer of Bcl2 from ER to MAM and mitochondria upon induction of apoptosis

**DOI:** 10.1038/s41419-021-03471-8

**Published:** 2021-02-15

**Authors:** Lisenn Lalier, Vincent Mignard, Marie-Pierre Joalland, Didier Lanoé, Pierre-François Cartron, Stéphen Manon, François M. Vallette

**Affiliations:** 1 CRCINA, INSERM, Université d’Angers, Université de Nantes, Nantes, France; 2 LaBCT, ICO, Saint Herblain, France; 3 Institut de Biochimie et de Génétique Cellulaires, UMR 5095 CNRS & Université de Bordeaux, Bordeaux, France

**Keywords:** Cancer, Apoptosis, Mitochondria

## Abstract

In this work, we have explored the subcellular localization of Bcl2, a major antiapoptotic protein. In U251 glioma cells, we found that Bcl2 is localized mainly in the ER and is translocated to MAM and mitochondria upon induction of apoptosis; this mitochondrial transfer was not restricted to the demonstrator cell line, even if cell-specific modulations exist. We found that the Bcl2/mitochondria interaction is controlled by TOM20, a protein that belongs to the protein import machinery of the mitochondrial outer membrane. The expression of a small domain of interaction of TOM20 with Bcl2 potentiates its anti-apoptotic properties, which suggests that the Bcl2–TOM20 interaction is proapoptotic. The role of MAM and TOM20 in Bcl2 apoptotic mitochondrial localization and function has been confirmed in a yeast model in which the ER–mitochondria encounter structure (ERMES) complex (required for MAM stability in yeast) has been disrupted. Bcl2–TOM20 interaction is thus an additional player in the control of apoptosis.

## Introduction

The processes of the early stages of apoptosis and its regulations are not completely understood although arguably crucial for the understanding of this process and thus the design of targeted therapies in cancer and degenerative diseases.

The elusive ER-mitochondria contacts called mitochondria-associated membranes (MAM) have been originally described as important in the reversible connection between ER lipids and mitochondria and in the ER/mitochondria calcium exchange^1^. Recently, MAM has been implicated in the control of several basic or pathological situations beyond lipid or calcium signaling^2^. MAM has been shown to be one of the sites of the formation of autophagosomes^3^ and linked to the onset of apoptosis^4^. The mechanisms that link MAM and apoptosis are multiple and currently not completely explored for most of them. Indeed, the best-studied pathway is the interaction between IP3R in the MAM and VDAC in mitochondria which has been shown to convey calcium-inducing apoptosis signals^5^. In recent work, we have identified sphingolipids modifications occurring in MAM, the mitochondria, and ER during the early phases of apoptosis that could be instrumental during the execution phase of the cell death program^6^.

The proteins of the Bcl2 family are structurally related regulators of apoptosis and function either as antiapoptotic agents (i.e., Bcl2, Bcl-xl, and Mcl-1) or proapoptotic proteins such as Bax or Bak in one hand and the BH3 only members (such as Bid, Bad, Bim…) in the other hand^7^. The antiapoptotic proteins inhibit apoptosis by binding the proapoptotic members. The main target of proapoptotic members of the Bcl2 family is the modification of the mitochondrial outer membrane (MOM) permeability (MOMP). The complex mechanisms of intra Bcl2 family interactions and the resulting control of mitochondrial permeabilization during apoptosis are still not completely understood despite the massive literature on the topic. Bcl2 can be found mainly in the ER and in mitochondria and there are some indications that its subcellular localization influences its function even at a distance of mitochondria (i.e., in the ER)^8,9^. It has been shown that the transmembrane domain (TM) of Bcl-xl targets a green fluorescent protein (GFP) to the MOM whereas Bcl2 TM addresses GFP to both mitochondrial and ER membranes^10^. It has been proposed that, during apoptosis, Bcl2 regulates ER-to-mitochondrion communication and thereby controls mitochondrial dysfunction and cell death by inhibiting proapoptotic proteins relocation to mitochondria and/or affecting calcium homeostasis via inositol 1,4,5-trisphosphate receptors (IP3R)^11^. Here, we have re-examined the subcellular localization of Bcl2 family members and found that Bcl2 is located in the ER in resting cells and is translocated to MAM and mitochondria upon the induction of cell death. We identified TOM20, a receptor of the MOM protein import, as the Bcl2 receptor at the early phase of apoptosis and explore the consequences of this interaction.

## Materials and methods

### Cell culture

The U251 cell line was grown in Dulbecco Modified Eagle Medium (4.5 g/L glucose) supplemented with 10% fetal calf serum, antibiotics (penicillin and streptomycin) and glutamine (Life Technologies, Carlsbad, CA) in 5% CO_2_ at 37 °C. The HeLa cell line was grown in Dulbecco Modified Eagle Medium (1 g/L glucose) + GlutaMAX supplemented with 10% fetal calf serum, antibiotics (penicillin, streptomycin) in 5% CO_2_ at 37 °C. Apoptosis was induced in cultures at 70% confluency with 0.5 μg/ml staurosporine (STS; Santa Cruz, Biotechnology, Heidelberg, Germany) or etoposide 50 µg/ml (Teva Classics, Paris, France). Plasmid transfection was performed with JetPrime reagent (Polyplus) according to the manufacturer’s instructions.

### Plasmids

GFP-Bcl2, GFP-Bcl2-cb5, and GFP-Bcl2-Maob were a gift from Clark Distelhorst (Addgene plasmid #17999, #18000, #18001)^12^. The plasmid encoding for GFP-tagged FATE1 was a gift from Enzo Lalli^13^ and the plasmid encoding for the RFP-tagged OMM-ER linker was a gift from György Hajnoczky^14^. The plasmids encoding for the peptides competing with Bcl2–TOM20 and Bcl2–IP3R interaction (respectively KRRSDPNFKNRLRERRKK and NVYTEIKCNSLLPLDDIVRV as identified in Rong et al.^15^) were synthesized by GeneCust (Luxembourg) by cloning the sequence encoding for the respective peptides in the pEGFP-C1 plasmid.

### Statistical analysis

All experiments were repeated as three independent replicates unless otherwise stated. The student *t* test was performed by the GraphPad Prism software.

### Microscopy

Transmission electron microscopy was done on cells fixed with 4% glutaraldehyde in PBS (pH 7.4) followed by a post-fixation with 2% OsO_4_. After dehydration in a graded series of ethanol, adherent cells were embedded in Epoxy resin, and thin sections (60–70 nm) were cut on a Reichert Ultracut E microtome and stained with uranyl acetate and lead citrate for observation at 80 kV under a JEM-1010 transmission electron microscope.

For microscopic analyses, cells were grown on glass cover-slips. When indicated, the cells were incubated with MitoTracker Red CMXRos (ThermoFisher Scientific) for 30 min at 37 °C, washed two times with PBS, and then were fixed in 4% paraformaldehyde for 15 min. The cells were washed with PBS and then mounted with Prolong antifade (Life Technologies) polymerizing solution, and observed under a microscope with a 63× objective (Zeiss with apotome).

### Subcellular fractionation

U251 cells from 40 Petri dishes (representing about 16 × 10^7^cells) were homogenized and subcellular fractionation prepared by differential centrifugation as previously described^6^. Briefly, after homogenization, unbroken cells and nuclei were removed, then an intermediate pellet was isolated from a fraction later centrifuged for ER and cytosol isolation, finally separated by ultracentrifugation (20,000*g* 30 min then 100,000*g* 1 h). The crude mitochondrial fraction (MF) was obtained by centrifugation of the previous resuspended pellet at 10,000*g* for 10 min. Crude MF was then layered on top of 30% Percoll medium and centrifuged at 95,000*g* for 30 min. After this centrifugation, a dense band of pure mitochondria (PM) was observed at the bottom of the tube and a diffused white band of MAM at the top of the tube. These fractions were collected with a Pasteur pipette, diluted, and then pelleted respectively at 6300*g* for 10 min and 100,000*g* for 1 h.

### Immunoblot analysis

For Western blot analysis of human proteins, 10 μg (fractions) or 50 μg (cell lysates) protein was loaded onto 8% or 12% sodium dodecyl sulfate-polyacrylamide gel electrophoresis (SDS-PAGE) and transferred onto Immobillion-P transfer membrane (Millipore, Darmstadt, Germany) for immunoblotting. Primary antibodies used were anti-TOM20 (sc-11415; Santa Cruz Biotechnology), anti-RTN3 (sc-374599; Santa Cruz Biotechnology), anti-GM130 (PA1-077; ThermoFisher Scientific), anti-Bcl2 (Ab131448; Abcam), anti-Mcl-1 (sc-819; Santa Cruz Biotechnology), anti-Bcl-xl (sc-56021; Santa Cruz Biotechnology), anti-Bax (4F11; Enzo), anti-Bak (556396; Pharmingen), anti-Bid (AF860; RnD Systems).

### Proximity ligation assay

Cells were seeded in 24-well plates onto glass sterile coverslips and allowed to attach overnight. The indicated treatment was applied, then cells were fixed by 4% paraformaldehyde. The labeling with antibodies was realized according to the manufacturer’s instructions (Duolink, Sigma-Aldrich). Primary antibodies used were anti-TOM20 (612278; Pharmingen), anti-Bcl2 (ab131448; Abcam), and anti-RTN3 (sc-374599; Santa Cruz Biotechnology). Image acquisition was performed with a Zeiss Apotome microscope (Zeiss Axiovert 200-M inverted microscope and AxioVision 4.6 program, Carl Zeiss Gbmh). Data were analyzed by the ImageJ 1.46r software and spots were quantified by the Object 3D Counter plugin. The images shown are representative of at least three independent experiments with at least two technical replicates by experiment. Quantification was made from at least 100 cells by conditions.

### Epitope mapping

Interaction of Bcl2 and TOM22 with TOM20 was performed using 12mer peptides and a two amino-acid frameshift, generating 68 peptides. Peptides were spotted on an Amino-PEG500-UC540 membrane using a MultiPep peptide synthesizer (Intavis AG, Cologne, Germany) at a loading capacity of 400 nmol/cm^2^. After synthesis the membrane was dried then the capped side-chains were deprotected by cleavage for 2 h with a cocktail containing 95% TFA, 3% triisopropyl, 2% H_2_O. TFA was removed and the membrane rinsed with dichloromethane, followed by ethanol and then H_2_O. The membrane was dried before use. STS-treated U251 cells were lysed in PBS-CHAPS 1% on ice for 30 min, then centrifuged 10 min at 16,000*g*. Membranes were saturated with 5% bovine serum albumin phosphate-buffered saline (BSA–PBS), and then incubated with 2 mg/ml protein extract. After which, it was washed three times with 1% BSA–PBS then positive spots were revealed as primary antibodies polyclonal anti-Bcl2 and TOM22 antibodies and secondary antibodies were revealed by the BM chemiluminescence kit (Roche Diagnostics). The binding intensities of Bcl2 or TOM22 for TOM20-derived peptides were determined by quantification using IP-Lab Gel software (Signal Analytics, Vienna, VA, USA) and converted to sequence-specific normalized units. The intensities obtained for each peptide covering a given amino acid were added and divided by the number of peptides.

### DEVDase activity in intact cells

U251 cells were transfected by the indicated plasmid (GFP- or RFP-tagged proteins or peptides) for 24 h, then plated in 6-well plates and treated by STS 0.5 µg/ml for 4 h. Cells were harvested and treated by the FLICA660 caspase 3/7 kit (Biorad) as recommended by the manufacturer and analyzed by a flow cytometer (Accuri C6). The mean FL4 fluorescence was measured in transfected cells (FL1- or FL2-positive cells) and normalized to the FL4 fluorescence in non-transfected cells.

### Viability assay

Cells were transfected with the indicated plasmids for 24 h, then plated in 6-well plates and treated with the indicated drugs (cytochalasin 2.5 µM 48 h, thapsigargin 10 µM 48 h, HBSS 48 h, CCCP 10 µM 48 h, TMZ 25 µM 72 h, RSL3 5 µM 72 h, ABT 737 0.1 µM 72 h, UMI77 1 µM 72 h). After treatment, cells were harvested by trypsinization and GFP-positive (FL1+) living cells were detected by To-Pro3 exclusion (FL4−) measured by flow cell analysis (BD Accuri C6 cytometer). The enrichment of GFP-positive living cells (FL1+/FL4−) in treated vs non-treated cells was calculated and the log of this value was designed as a protection index. The graph shown is representative of four independent experiments.

### Yeast experiments

The strain W303-1A (*mat a*, *ade1*, *his3*, *leu2*, *trp1*, and *ura3*) was used in all the experiments. The strain W303/*Δmdm34* was obtained by the amplification of a Mdm34::kanMX4 fragment from genomic DNA of the strain BY4742/*Δmdm34* from Euroscarf collection. The amplified fragment was used to transform W303-1B cells by homologous recombination. Transformed cells were selected on the basis of geneticin resistance and verified by a polymerase chain reaction. The destabilization of ERMES in the W303/*Δmdm34* was reported previously^16^.

The construction of pYES3-BaxP168A and the pYES2-BclxL plasmids was described previously^17^. These plasmids express full-length and untagged human Bax or Bcl-xL under the control of a GAL1/10 promoter.

The pYES2-Bcl2 (full length), pYES2-Bcl2-Maob, and pYES2-Bcl2-cb5 were constructed by amplifying the Bcl2 construction (without the GFP part) from the GFP-Bcl2 plasmids (Addgene) and cloning between the HindIII/EcoR1 sites of pYES2/CT (with a stop codon to eliminate the His6-V5 tag). The pYES3-yTom20 was constructed by amplifying the yTom20 gene from yeast genomic DNA and cloning between the BamH1/Not1 site of pYES3/CT plasmid (with a stop codon to eliminate the His6-V5 tag). The pYES3-hTom20 was constructed by amplifying the hTom20 sequence from a mCherry-Tom20 construction (gift from Dr. Manuel Rojo, IBGC) and cloning between the EcoR1/Not1 sites of pYES3/CT (with a stop codon to eliminate the his6-V5 tag). The pESC-GFP-TBI was constructed by amplifying the GFP-TBI sequence from the pEGFP-pepTOM20 (TBI) plasmid and cloning in the BamH1 site of the pESC-His3 plasmid (with a stop codon to eliminate the c-myc tag).

All the constructions were verified by sequencing both strands.

Yeast cells were grown at 28 °C under rotary agitation in 5 l Erlenmeyer flasks containing 1 l of YNB medium (Yeast Nitrogen Base (Difco) 0.67 g/l, ammonium sulfate 5 g/l, potassium phosphate 1 g/l, Drop Mix 2 g/l, adequate auxotrophic markers 0.1 g/l, pH 5.2), supplemented with 2% dl-lactic acid as the carbon source until the cell density reached ~10^7^ cells/ml. The expression of Bcl2 variants, yeast Tom20, human Tom20, GFP-TBI, Bax-P168A, and Bcl-xL was induced by adding 0.5% galactose. After 14 h, 4 × 10^7^ cells were used to prepare whole-cell extracts, and the rest of the cultures was used to isolate mitochondria. Both methods are detailed elsewhere^18^. Rapidly, mitochondria isolation was performed as follows: whole-cell lysates were loaded on top of a 25–65% Optiprep gradient; after 16-h centrifugation at 105,000 × *g*, gradients were fractionated in 10 fractions that were analyzed by SDS-PAGE and western-blotting against Pgk1 (cytosolic marker) and Por1 (mitochondrial marker) defined PMS and MFs, respectively.

Protein concentration was measured by the Lowry method. Totally, 50–100 µg of each fraction was loaded on 12.5% SDS-PAGE, transferred onto nitrocellulose (Amersham Protran), and blotted with the following antibodies: Bcl-x (rabbit monoclonal, Abcam 32370), Bcl2 (rabbit polyclonal, Abcam 7973), Bax (mouse monoclonal, Santa-Cruz Sc-20067), GFP (mouse monoclonal, Roche 11814460001), human Tom20 (rabbit polyclonal, Thermofisher PA5-86739), yeast Tom20 (rabbit polyclonal, a gift from Dr. Carla Koehler, UCLA), yeast Por1 (mouse monoclonal, Thermofisher 459500), yeast Pgk1 (mouse monoclonal, Thermofisher 459250), HRP-conjugated anti-mouse and anti-rabbit IgG (Jackson Immunoresearch). Blots were revealed by ECL (Luminata Forte, Millipore), digitally acquired (G-Box, Syngene), and quantified with Image J.

## Results

### Bcl2 is localized in ER in resting cells but becomes mitochondria-bound at the early stage of apoptosis

**Fig. 1:**
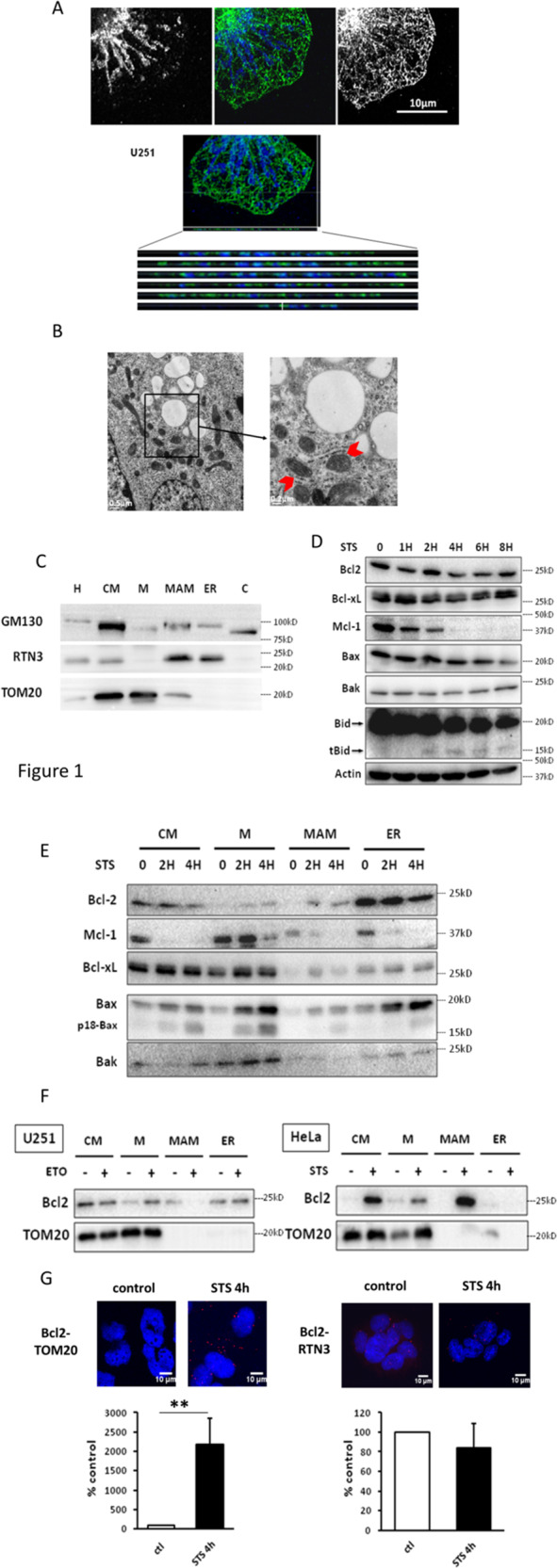
Bcl2 localization during the early phase of apoptosis. **A** Confocal analysis of MFN1 (blue) and RTN3 (green) shows the close proximity of mitochondria and ER in U251 cells. **B** Electron microscopy in U251 cells enables the visualization of MAM, formed by the juxtaposition of ER and mitochondria (red arrowheads). **C** Cell fractionation is realized in U251 cells and fractions are analyzed by western blot (GM130: Golgi protein, RTN3: ER protein, TOM20: mitochondria protein) (H: homogenate, CM: crude mitochondria, M: pure mitochondria, C: cytosol). The blots are representative of three independent fractionation experiments. **D** U251 cells were treated by STS and total lysates are analyzed by western blot. **E** U251 cells treated by STS for 0, 2 and 4 h are fractionated as in (**C**). Fractions are analyzed by western blot. **F** U251 cells were treated by etoposide for 24 h and HeLa cells by STS for 4 h. Cell fractionation was realized as in (**E**) and Bcl2 localization was analyzed by western blot. The blots shown in **D**–**F** are representative of three independent experiments. **G** Bcl2–TOM20 and Bcl2–RTN3 interactions were observed by PLA in U251 treated by STS.

To gain knowledge on the implication of MAM in the early steps of apoptosis, we used STS-treated U251 glioma cells. We measured the kinetics of caspase 3 activation and observed that caspase 3 activity reached a plateau 4 h after STS treatment (Fig. S1A). We defined as “early phase of apoptosis” the time frame during which STS induces a modification of the mitochondrial network without altering the mitochondrial membrane potential (Fig. S1B), while caspase 3 activation (Fig. S1A) and cytochrome c release begin and no cell death occurs, as previously described by Mignard et al.^6^. MAM was visualized in resting U251 by confocal and electron microscopic analyses (Fig. 1A, B) and analyzed by subcellular fractionation (Fig. 1C) to confirm that the crude mitochondria fraction (CM) is composed of mitochondria-associated to MAM; after percoll-gradient centrifugation, MAM were separated from PM (M, Fig. 1C), as shown by the segregation of TOM20 in the mitochondria fraction (M) and of RTN3 in the ER fraction, whereas MAM expresses both proteins. VDAC–IP3R interaction is commonly used to monitor MAM formation^19^; we observed an increase in VDAC–IP3R interaction in the early phase of apoptosis (Fig. S1C).

We analyzed the expression of several proteins from the Bcl2 family in total cell lysates from 0 to 8 h following STS treatment. Their expression was differently affected during this time course of STS treatment (Fig. 1D). Mcl-1 was rapidly degraded at the early phase of apoptosis (i.e., 2–4 h) while Bax was degraded at later stages (i.e., 8 h). The BH3-only protein Bid was cleaved into a lower molecular weight band (t-Bid) after 2 h post treatment as previously described^20,21^. In contrast, the expression of Bcl2, Bcl-xl, and Bak remained constant. Next, the protein content in subcellular fractions of STS treated U251 cells (during early apoptosis as described above, 2–4 h post treatment) was analyzed as described by Wieckowski et al.^22^; the characterization of the fractions and markers expression in resting cells and at the early phase of apoptosis is detailed in Mignard et al.^6^. As shown in Fig. 1E, Bcl2 was mainly located at the ER in resting U251 cells. However, upon induction of apoptosis, as early as 2 h, Bcl2 was found in the MAM and in the M fraction. In contrast, Mcl-1 was found in all the fractions but was degraded much slower in the M fraction than in the other subcellular compartments. Bcl-xl was found mostly in CM and M fractions and its localization was not significantly altered by the treatment. The proapoptotic protein Bax, mostly cytosolic in untreated U251 (not shown) was targeted to all the fractions upon STS treatment, together with the appearance of the shorter, cleaved p18-Bax form generated at the onset of apoptosis^23^. In contrast, Bak, another proapoptotic member of the Bcl2 family, was mostly present in the mitochondria (M) and its localization was not altered by the treatment. These results show that, in U251 resting cells, Bcl2 is associated with ER rather than mitochondria; at the early phase of apoptosis, Bcl2 partly translocates to MAM and mitochondria. To avoid the possibility that Bcl2 partial relocalization to the MAM and mitochondria was treatment- or cell line-dependent, we repeated the subcellular fractionation in etoposide-treated U251 cells and with STS-treated HeLa cells (Fig. 1F). Bcl2 relocalization to MAM and mitochondria is reproduced in these experimental conditions, although very little Bcl2 was found in the ER in resting HeLa cells. More accurately, it appears that Bcl2 shifts from the MAM to the mitochondria in etoposide-treated U251 cells, rather than from ER to MAM and mitochondria, whereas Bcl2 expression disappears from the ER in STS-treated HeLa cells and increases in the MAM and mitochondria fractions. Altogether, these results suggest that in resting cells Bcl2 is not/little associated with mitochondria but with ER and/or MAM in U251 and HeLa cells. In the early phase of apoptosis defined upper, Bcl2 shifts toward MAM and mitochondria.

Motz et al.^24^ have suggested that Bcl2 could interact with TOM20. We thus studied the interaction between Bcl2 and TOM20 in glioma cells upon induction of apoptosis, in control and STS-treated U251 cells, by in situ proximity ligation assay (PLA) (Fig. 1G). As illustrated in Fig. 1G, Bcl2–TOM20 interaction happened in early apoptosis, concomitant to MAM increase (observed by VDAC-IP3R increase, Fig. S1C). Bcl2–RTN3 interaction was measured by PLA as a control and was not modified by STS. The images shown are representative from the cells observed in control and treated cells. The number of PLA spots was quantified by the ImageJ software and normalized by the number of cells in the field as described in the section “Materials and methods”.

We conclude that the interaction between Bcl2 and mitochondria occurs at an early stage of apoptosis and is concomitant to its interaction with TOM20.

### Bcl2 forced localization to ER does not affect its interaction with TOM20 at the onset of apoptosis

**Fig. 2:**
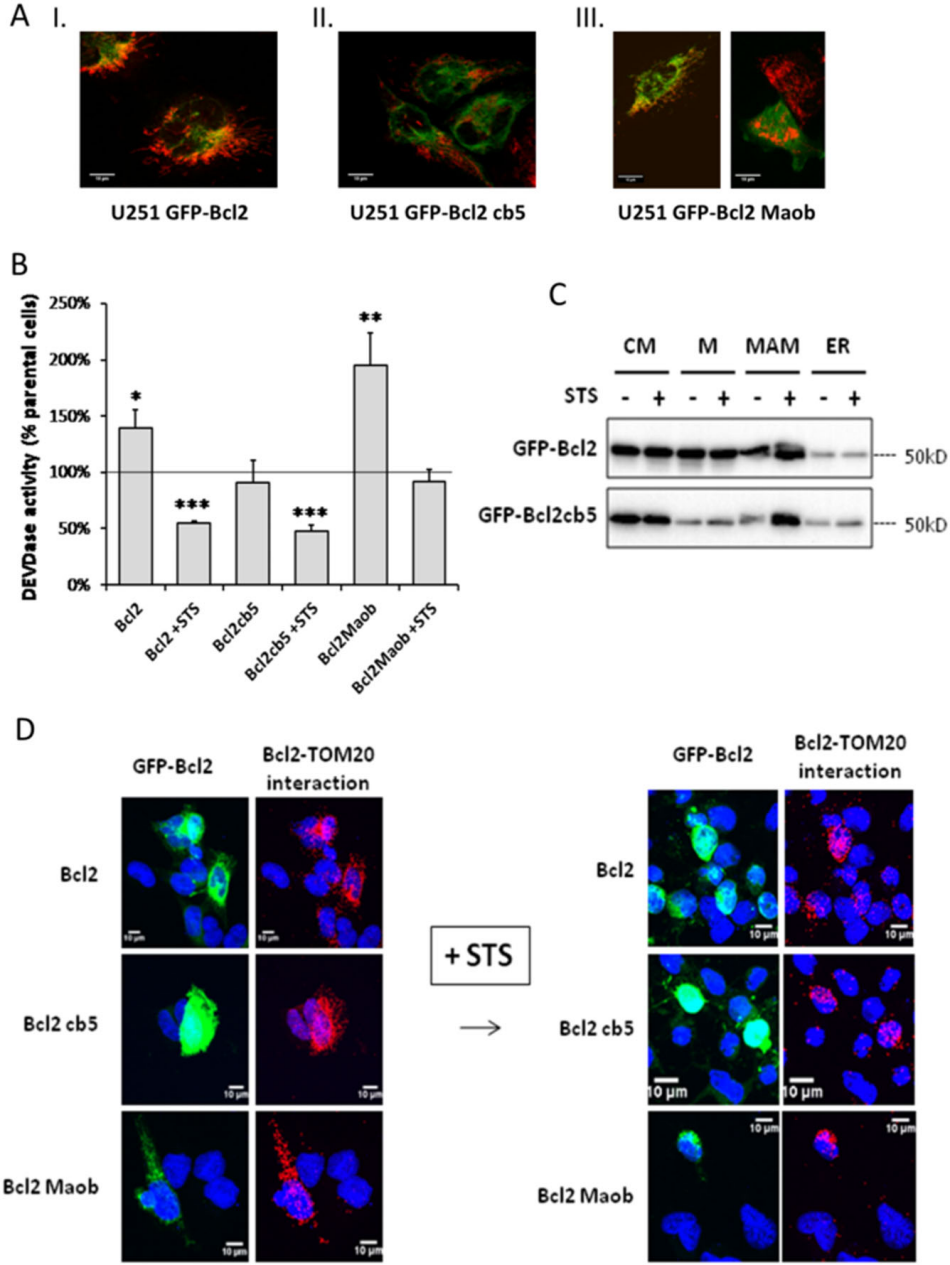
Bcl2 forced targeting to ER does not alter its function nor interaction with TOM20. **A** U251 were transfected by GFP-Bcl2, GFP-Bcl2cb5 (ER targeting), or GFP-Bcl2Maob (mitochondrial targeting) and mitochondria were stained by mitotracker Red CMXRos. **B** U251 cells were transfected as in (**A**) and cells were treated by STS for 4 h. DEVDase activity was measured in protein extracts, normalized by the protein concentration, and corrected by the activity measured in non-treated cells. **C** Stably transfected U251 cells were treated by STS and cell fractionation was performed as described in Fig. 1. Bcl2 localization was analyzed by western blot. The blots shown are representative from three independent experiments. **D** Transfected U251 cells were treated by STS and Bcl2–TOM20 interaction was measured by PLA.

We further investigated the role of Bcl2 relocation from ER/MAM to mitochondria using the chimeric, GFP-tagged Bcl2 targeted constructs in which the C-terminal transmembrane domain of Bcl2 was replaced by that of Maob for mitochondrial targeting or of cb5 for ER targeting^12,25^. Contrary to the rest of the results shown in this article, results shown in Fig. 2 were obtained with cells overexpressing Bcl2 through the transfection of plasmids expressing an either form of targeted Bcl2. In U251, GFP-Bcl2 exhibited a partial mitochondrial distribution (Fig. 2A I, mitochondria are stained in red), while, as expected, GFP-Bcl2cb5 did not colocalize with mitochondria (Fig. 2A II). In GFP-Bcl2Maob transfected cells, GFP-Bcl2Maob exactly colocalized with mitochondria (Fig. 2A III, left picture), but we noticed that most of the GFP-expressing cells were actually dying (Fig. 2A III, right picture). Transfected cells were treated by STS and DEVDase activity was measured by flow cytometry in transfected cells (FL1+ cells) versus non-transfected cells (FL1− cells) in the same wells as described in the “Material and methods” section (Fig. 2B). As already described^12^, the transient transfection of GFP-Bcl2, and even more that of GFP-Bcl2Maob, induced apoptosis whereas Bcl2cb5 does not (Fig. 2B). Nevertheless, cells overexpressing GFP-Bcl2 were more resistant to STS than their non-transfected counterparts. GFP-Bcl2cb5 also significantly protected cells from STS-induced apoptosis. However, the expression of GFP-Bcl2Maob did not reduce the sensitivity of the cells to STS-induced apoptosis. Early studies have shown that the Bcl2cb5 fusion specifically targets the ER and protects cells from most but not all apoptosis inducers^26^. In our hands, it appeared that ER-targeted Bcl2 was as efficient as Bcl2wt in the inhibition of STS-induced apoptosis, whereas the overexpression of mitochondria-targeted Bcl2 in U251 cells was not protective; moreover, the transient overexpression of Bcl2 or its mitochondria-targeted construct both induce apoptosis.

**Fig. 3:**
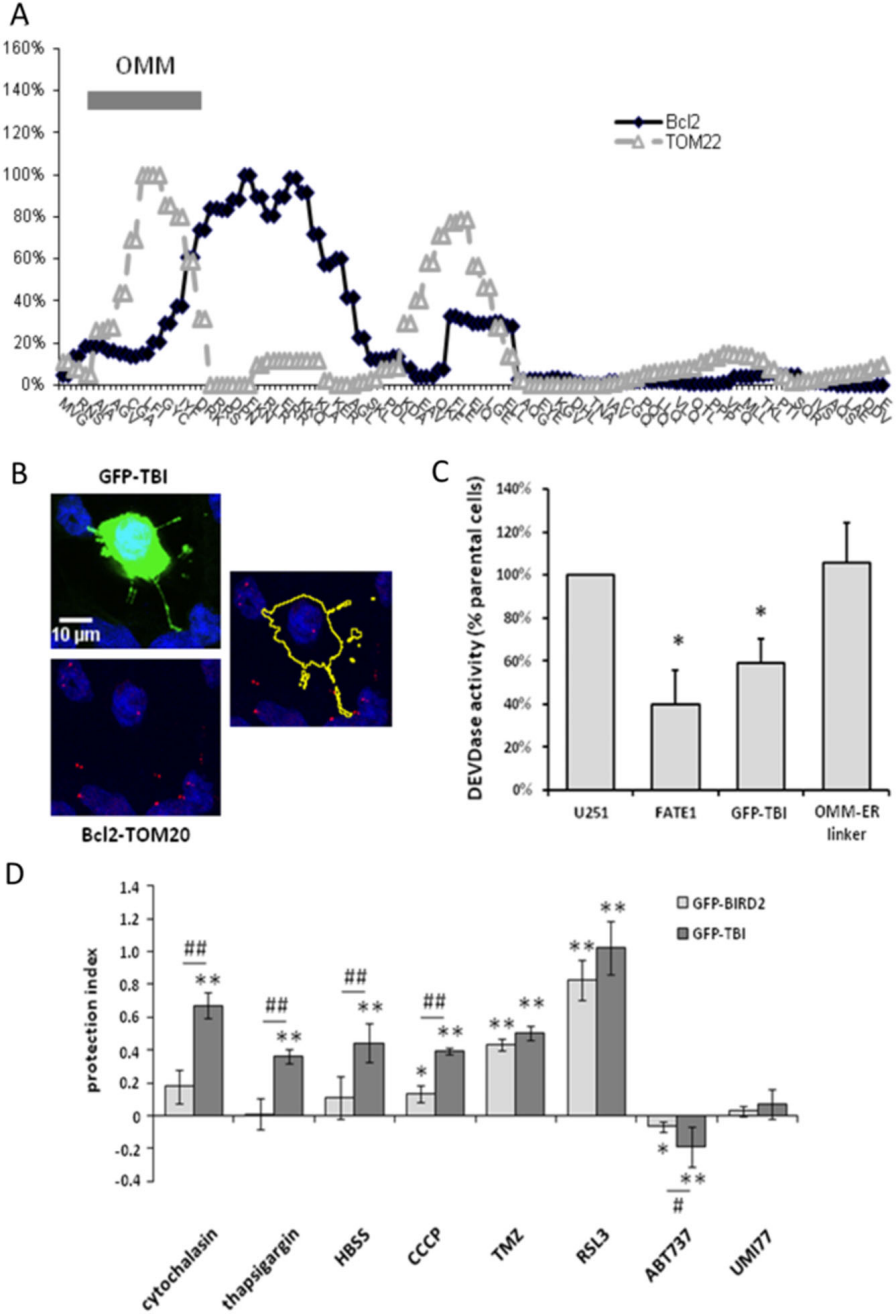
Bcl2–TOM20 interaction is inhibited by a competitive peptide. **A** Epitope mapping suggests a major putative sequence of TOM20 interacting with Bcl2. The intensity of TOM20 interaction with Bcl2 is represented by the black diamonds and the gray triangles represent the intensity of TOM20 interaction with TOM22 along the TOM20 sequence. (OMM: outer mitochondrial membrane). **B** TBI-GFP was expressed in U251 cells then treated by STS (4 h). Bcl2–TOM20 interaction was measured by PLA. **C** U251 cells were transfected by a plasmid expressing the fluorescent proteins or peptides indicated. Cells were treated by STS and DEVDase activity was measured in the transfected cells by flow cytometry and expressed as the percentage of the activity measured in non-transfected cells. **D** GFP-tagged peptides were expressed in U251 cells later treated by the indicated drugs (see “Material and methods” for details). The protection index compared to the cell death induced in non-transfected cells was indicated on the graph (mean ± sd; *p < 0.05 and **p < 0.01 vs. non-transfected cells, ^#^*p* < 0.05 and ^##^*p* < 0.01 between TBI-GFP and BIRD2-GFP).

Next, we questioned the ability of organelle-targeted Bcl2 to shift from an organelle to the other during apoptosis induction. In order to perform cell fractionation, we had to select and amplify the transfected cells for several weeks. Cell fractionation is the only experiment performed with stably expressing, antibiotics-selected U251 cells, which produced slightly different results in Bcl2 localization. Indeed, long-term ectopic protein expression, or more probably antibiotics treatment, stressed the cells, resulting in a large amount of mitochondria-located Bcl2, even in the cells transfected with GFP-Bcl2cb5 (Fig. 2C). Despite this difference in the basal balance between mitochondria- and ER-located Bcl2, STS still induces an increase in the amount of Bcl2 in the MAM fraction. Of note, the toxicity of Bcl2Maob made it impossible to induce the long-term expression of Bcl2Maob required for cell fractionation. Last we studied the interaction between TOM20 and Bcl2 in the chimeric-protein expressing cells by PLA. Bcl2–TOM20 interaction was observed in the cells expressing either Bcl2 variant upon STS-induced apoptosis (Fig. 2D). In contrast to endogenous Bcl2 (Fig. 1G), GFP–Bcl2–TOM20 interactions were already observed in transfected non-treated cells, probably because of Bcl2 large overexpression (Fig. 2D). As a conclusion, our results suggested that Bcl2 localization to the ER was favored by the cb5 tag, but the ER signal did neither prevent its translocation to MAM and mitochondria nor its antiapoptotic function. Bcl2 interacts with TOM20 even when Bcl2 C-terminus is replaced by an organelle-specific targeting sequence.

### Bcl2–TOM20 interaction is inhibited by a competitive peptide

We used an epitope mapping strategy to identify the regions of TOM20 implicated in the interaction with Bcl2 (see “Material and methods”). As shown in Fig. 3A, we identified a major putative interaction region which is located at the N-terminus cytosolic side of the protein and which is encompassing the positive charge membrane sequence of TOM20 (Fig. S2A). Interestingly, this region was different from that implicated in TOM20 interaction with TOM22 (Fig. 3A)^27^ and domains interacting with imported mitochondrial proteins (Fig S2A). The sequence identified (KRRSDPNFKNRLRERRKK), called **T**OM20–**B**cl2 **i**nteraction sequence (TBI), had no sequence homology with any other known proteins than TOM20 in different species (including *Saccharomyces cerevisiae*). Of note, the reverse analysis of Bcl2 domains interacting with TOM20 by similar methods did not allow the identification of a unique linear sequence of interaction with TOM20 (Fig. S2B). Thus, Bcl2 showed a unique pattern of interaction with TOM20, different from the protein import pathway into mitochondria as previously suggested by Motz et al.^24^. We constructed a chimeric protein with a TBI sequence coupled to the C-terminus of GFP (GFP-TBI) to explore its putative effects on Bcl2–TOM20 interaction. As illustrated in Fig. 3B, in U251 cells treated by STS, Bcl2–TOM20 interaction was efficiently inhibited in GFP-TBI expressing cells. Next, we investigated the possible contribution of Bcl2–TOM20 interaction to the control of mitochondria–ER contacts. We compared the sensitivity of U251 cells to STS-induced apoptosis when expressing either a mitochondria–ER spacer (FATE1)^13^ or mitochondria–ER tether (OMM-ER linker)^14^ and compared it to GFP-TBI (Fig. 3D). FATE1 actually protected the cells from apoptosis whereas no sensitization was observed with the OMM–ER linker. In contrast, cells expressing GFP-TBI were less sensitive to STS-induced apoptosis than parental cells (Fig. 3C). This protective effect was not observed in the H358 lung cancer cell line in which no detectable expression of Bcl2 was observed (Fig. S3A). This suggests that Bcl2–TOM20 interaction is implicated in the control of apoptosis. Of note, VDAC-IP3R interaction was not inhibited in cells expressing GFP-TBI (Fig. S3B). Thus MAM was not disrupted by GFP-TBI, and FATE1 or OMM-ER linker expression did not alter Bcl2–TOM20 interaction (Fig. S3C, D). These results suggest that Bcl2–TOM20 interaction is not involved in the stability of apoptotic ER–mitochondria contacts. Bcl2 also interacts with IP3R, thereby interfering with the ability of Ca^2+^ to transfer from the ER to mitochondria. This interaction is inhibited by the synthetic peptide BIRD2, derived from IP3R, which targets the BH4 domain of Bcl2 and induces pro-apoptotic Ca^2+^ signals^15^. We used the sequence of IP3R interacting with Bcl2 in BIRD2, coupled it to GFP, and named it GFP-BIRD2. GFP-TBI or GFP-BIRD2 were expressed in U251 cells. We tested the sensitivity of transfected cells to a panel of drugs with various death-inducing mechanisms (Cytochalasin, an actin polymerization inhibitor; Thapsigargin, a SERCA inhibitor; serum-deprived culture media, an autophagy inducer; CCCP, a mitophagy inducer; Temozolomide, a DNA methylating agent and RSL3, a ferroptosis inducer) and two BH3 mimetics (ABT737, Bcl2 inhibitor and UMI77, and Mcl-1 inhibitor). As shown in Fig. 3D, GFP-TBI expression significantly decreased cells sensitivity to all the drugs and treatments but sensitized them to the BH3 mimetic ABT737 and had no effect towards UMI77. In the conditions used, GFP-BIRD2 protected the cells against CCCP, TMZ, and RSL3, but was not equivalent to GFP-TBI. Thus, the inhibition of the Bcl2–TOM20 interaction appears to be implicated in a large variety of cell death inductions.

### Bcl2 interacts with TOM20 in yeast

**Fig. 4:**
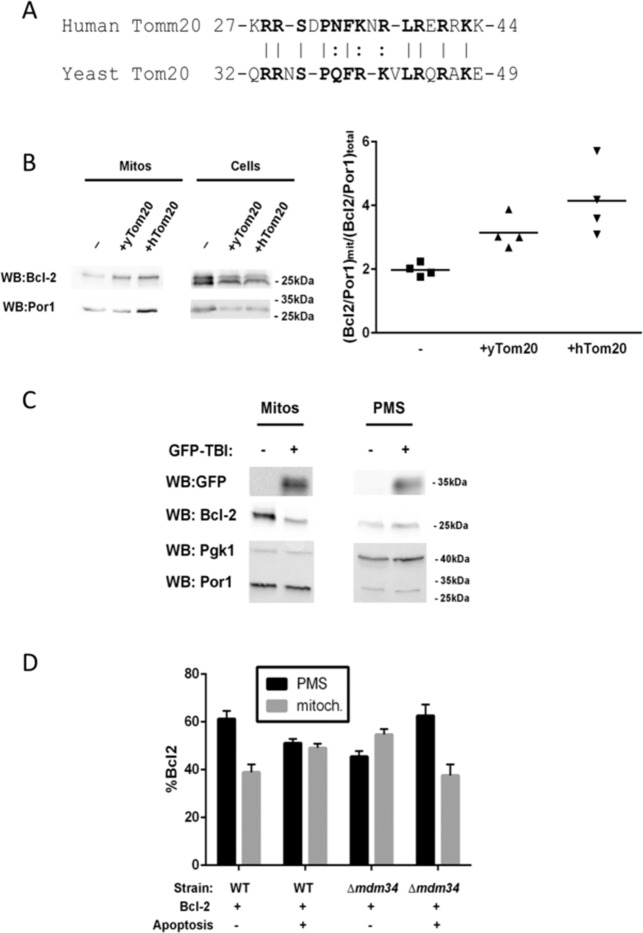
Bcl2 interacts with TOM20 in yeast. **A** Sequence homology between human and yeast TOM20 in the TBI sequence. **B** Yeast or human TOM20 expression wasinduced in *S. cerevisieae* together with human Bcl2. After 14 h, whole-cell extracts and isolated mitochondria were analyzed for Bcl-2 content by western blot. Mitochondrial porin (Por1) was used as a mitochondrial loading control. The ratio Bcl2/Por1 was quantified for each blot and the mitochondrial ratio was compared to the total ratio. **C** GFP-TBI expression was induced in yeast cells expressing human Bcl2, for 14 h, and isolated mitochondria and post-mitochondrial supernatants (PMS) were analyzed for Bcl-2 content. Mitochondrial porin (Por1) and Phosphoglycerate Kinase (Pgk1) were used as mitochondrial and cytosolic markers, respectively. **D** The expression of the constitutively active mutant BaxP168A was induced in wild-type yeast cells expressing human Bcl2 or the Δmdm34 mutant expressing human Bcl2. Mitochondria and PMS were analyzed for Bcl-2 content by western blotting.

No Bcl2 family homologs are constitutively expressed in yeast, but still Bax can kill S.cerevisae and Bcl2 inhibit this death^28^. Thus yeast has been an interesting model to study the constitutive cellular machineries and partners common in Eukaryotes that are implicated in cell death. As illustrated in Fig. 4A, human TOM20 and its yeast homolog share a strong sequence homology at the TBI site with a remarkable conservation of the juxtamembrane positive charges. We overexpressed yeast and human TOM20 in the *S. cerevisiae* W303D strain expressing human Bcl2 (Fig. S4A) and measured Bcl2 localization by cell fractionation (Fig. 4B). The mitochondrial localization of Bcl2 was increased in yeast transformed by yTOM20 or hTOM20, human TOM20 being more efficient in Bcl2 mitochondrial addressing than its yeast homolog (Fig. 4B). Of note, no significant change in Bcl-xl localization was observed in the same conditions (Fig. S4B). We then expressed the TBI-GFP in Bcl2-expressing *S. cerevisae*. As observed in mammalian cells, the expression of the competing peptide inhibits Bcl2 targeting to mitochondria. Similar situation was found in either the Bcl2 wild-type (Fig. 4C) or the C-terminally modified Bcl2, as described in Fig. 2 (Bcl2 C-terminus replaced by the mitochondrial targeting sequence of Maob or by the ER-targeting sequence of cb5, Fig. S4C).

We have previously shown that the MOMP happening during apoptosis in mammalian cells can be mimicked in yeast by the expression of an active Bax (namely Bax P168A)^29^. Figure 4D shows that Bcl2 mitochondrial localization (gray bars) is increased when apoptosis is induced. We then deleted mdm34, a gene coding for a protein of the ERMES complex, implicated in MAM stabilization^16^. MDM34 deletion raised the mitochondrial localization of Bcl2, suggesting that Bcl2 is actively pulled out of the MOM through the MAM in resting yeast cells. When apoptosis is induced, the mitochondrial Bcl2 lowers, showing that Bcl2 mitochondrial transfer during apoptosis requires MAM integrity.

## Discussion

Bcl2 has been found in many subcellular membranes but mostly in the endoplasmic reticulum and mitochondria. Here, we show that Bcl2 is also located to MAM and that it migrates to mitochondria during early apoptosis, i.e., before the disruption of the mitochondrial network (Fig. 1). The mitochondrial transfer of Bcl2 relies on Bcl2–TOM20 interaction. TOM20 is a protein inserted in the MOM and belongs to the protein mitochondrial import system. An interaction between Bcl2 C-terminus and yeast TOM20 was previously reported in an acellular system^24^. The interaction we report here is not dependent on Bcl2 C-terminus, since the replacement of this sequence by the targeting sequences of Maob or cb5 did not alter Bcl2 interaction with TOM20 (Fig. 2D). Moreover, in yeast, the mitochondrial localization of human Bcl2 but not that of Bcl-xl was altered by TOM20 expression (either human or yeast TOM20) (Fig. 4B and Fig. S4B). Last, we identified a putative interaction sequence between TOM20 and Bcl2 in TOM20 sequence; the expression of a peptide corresponding to this sequence actually inhibits the interaction between Bcl2 and TOM20 in human cells, and the mitochondrial translocation of Bcl2 in yeast (Fig. 4C).

The expression of the mitochondria-targeted GFP-Bcl2Maob chimeric protein does not protect U251 cells from the STS-induced apoptosis but rather increases their sensitivity (Fig. 2B). The proapoptotic effect of GFP-Bcl2Maob has already been reported^12^. This somehow appears conflicting with the undeniable function of Bcl2 as an inhibitor of Bax/Bak-dependent MOMP. Yet it was shown that Bcl2 family antiapoptotic members, despite protecting cells from apoptosis, prime them to apoptosis through their interaction with Bax or Bak^29–31^. Bcl2 mitochondrial localization through TOM20 might thus participate to Bax-induced apoptosis as a vehicle^32^, taking Bax close to its mitochondrial apoptotic receptor TOM22 (ref. ^33^) which is known to interact with TOM20 (ref. ^27^). The physical proximity between TOM20 and TOM22, joined to the participation of Bcl2 to Bax conformation switch^34^, might participate to the initial induction of massive MOMP observed in apoptosis. This is consistent with the fact that the inhibiting peptide GFP-TBI protects cells from most of the death inducers we tested, whereas it sensitizes the cells to the death induced by the BH3 mimetic ABT737 (Fig. 3D). In contrast, the inhibition of Bcl2–Bax interaction (by ABT737) does not inhibit Bcl2–TOM20 interaction (Fig. S5).

MAM are crucial membranes juxtaposed to the mitochondria, mostly constituted by ER–mitochondria apposition, whose role in apoptosis is raising since their regulation of Bax and Bak activity was demonstrated^35^. In the early apoptosis, biological material is exchanged between ER and mitochondria, among which ceramides^6^ and proteins, including Bax^16^ and Bcl2 as described in this study. The regulation of MAM dynamics, defining the proximity between organelles and probably the ability of molecules to transfer from one organelle to the other, thereby appears as a new player in the regulation of MOMP. In yeast, a single protein complex, namely ERMES, is responsible for MAM stability, making it a good model to investigate the role of MAM in the mechanism. As MDM34 deletion impairs Bax mitochondrial localization and apoptotic activity^16^, it also disturbs Bcl2 localization. In resting conditions, MAM destabilization increases mitochondrial Bcl2 (Fig. 4D), suggesting that MAM enable Bcl2 active retrotranslocation from the mitochondria to the ER. Bax is retrotranslocated from the mitochondria by Bcl2, Bcl-xl, and Mcl1 in resting cancer cells^36^. Our results suggest that Bcl2 also translocates from the mitochondria to the ER in a MAM-dependent fashion, maybe together with Bax. This observation might nevertheless be an artifact due to the yeast model since yeast does not naturally express Bcl2; indeed, the targeted expression of Bcl2Maob or Bcl2cb5 turned out to be difficult. Further investigations in mammalian cells should therefore confirm this active retrotranslocation in resting conditions. When apoptosis is induced, Bcl2 mitochondrial translocation requires the interaction with TOM20, MAM disruption thus induces the decrease of Bcl2 mitochondrial localization. MAM, therefore, confirm their central role in the regulation of the MOMP, regulated by the Bcl2 family.

Our study thus further identifies proteins of the TOM complex as major interaction partners of the Bcl2 family in the control of MOMP during apoptosis. The role of MAM in the regulation of this interaction network is certainly a new intermediate in the early steps of apoptosis.

## Supplementary information

Supplementary figures
