# Supplementary material for: TOM20-mediated transfer of Bcl2 from ER to MAM and mitochondria upon induction of apoptosis
Source: Cell Death Dis. 2021 Feb 15;12(2):182. doi: 10.1038/s41419-021-03471-8 (PMC7884705; doi:10.1038/s41419-021-03471-8)
Supplement: Supplementary file 1 — Supplementary figures [file 41419_2021_3471_MOESM1_ESM.pdf]

Supplementary figures

Figure S1: A) U251 cells were treated by STS for the indicated time. Protein extraction was realized and DEVDase activity was measured and normalized to the protein content. Data represent mean+/-sd from three independent experiments. B) U251 cells were spotted onto glass coverslips, then treated by STS for 4h. Mitotracker Red CMXRos was added for the last 15mn. Cells were fixed and observed with a confocal microscope. C) U251 cells were treated as in B, then fixed and VDAC-IP3R interaction was observed by PLA. The image is representative for STS-treated cells. Spots were quantified by FiJi (3D Object Counter plugin) and normalized by the number of cells in the field. The graph represents the percentage of spots compared to control cells.

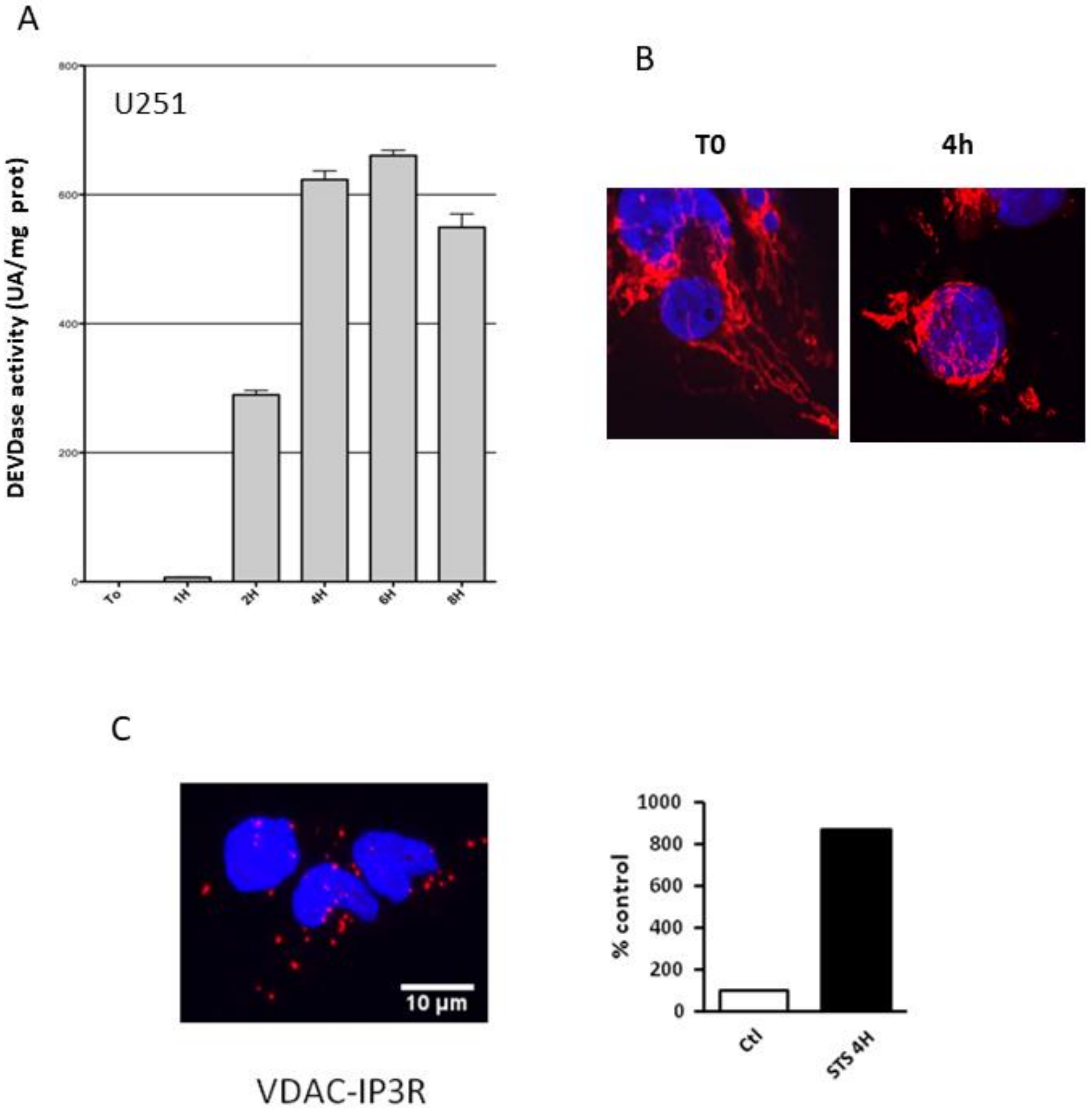
A
U251
DEVDase activity (UA/mg prot)
800
600
400
200
0
To
1H
2H
4H
6H
8H
B
T0
4h
C
10 µm
VDAC-IP3R
% control
1000
800
600
400
200
0
Ctl
STS 4H

Figure S1

Figure S2: A) The schematic structure of TOM20 is represented to depict the different functional domains: the transmembrane region, the juxtamembrane Bcl2-binding domain, the TPR core and the acidic tail. The Bcl2-binding region identified by epitope mapping is underlined in TOM20 sequence indicated below the molecule. B) An epitope mapping was realized by spotting the peptides corresponding to Bcl2 sequence on a membrane later incubated with STS-treated U251 proteins. TOM20 binding was revealed by the use of an anti-TOM20 antibody. Signal was quantified for each aminoacid and represented on the graph. Bcl2-homology domains (BH1-4) are figured on the graph.

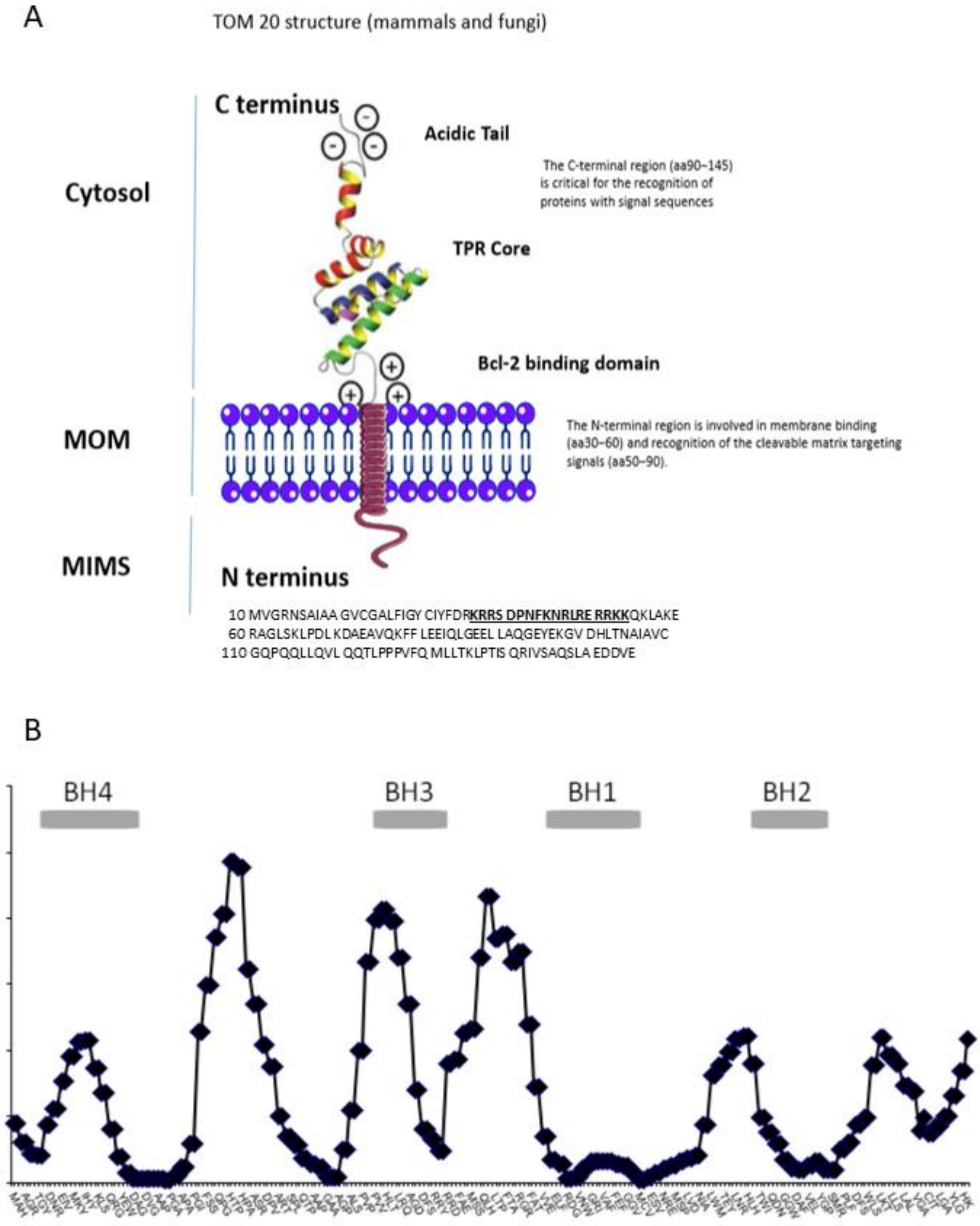
A
TOM 20 structure (mammals and fungi)
Cytosol
MOM
MIMS
C terminus
Acidic Tail
The C-terminal region (aa90–145) is critical for the recognition of proteins with signal sequences
TPR Core
Bcl-2 binding domain
The N-terminal region is involved in membrane binding (aa30–60) and recognition of the cleavable matrix targeting signals (aa50–90).
N terminus
10 MVGRNSAIAA GVCGALFIGY CIYFDRKRRS DPNFKNRLRE RRKKQKLAKE
60 RAGLSKLPDL KDAEAVQKFF LEEIQLGEEL LAQGEYEKGV DHLTNAIAVC
110 GQPQQLLQVL QQTLPPPVFQ MLLTKLPTIS QRIVSAQSLA EDDVE
B
BH4
BH3
BH1
BH2
600000
500000
400000
300000
200000
100000
0

Figure S2

Figure S3: A) Lung cancer cells (H358) expressing no Bcl2 were transfected with a plasmid encoding for GFP-TBI. Cells were treated by STS for 4h and DEVDase activity was measured by flow cytometry as described in material and methods. B) VDAC-IP3R interaction was observed in U251 cells expressing GFP-TBI treated by STS by PLA (red spots). Transfected cells appear as GFP-positive. The image is representative for the cells observed in three independent experiments. C) Bcl2-TOM20 interaction was observed in U251 cells expressing FATE1 treated by STS by PLA (red spots). Transfected cells appear as GFP-positive. The image is representative for the cells observed in three independent experiments. D) Bcl2-TOM20 interaction was observed in U251 cells expressing OMM-ER linker treated by STS by PLA (green spots). Transfected cells appear as RFP-positive. The image is representative for the cells observed in three independent experiments.

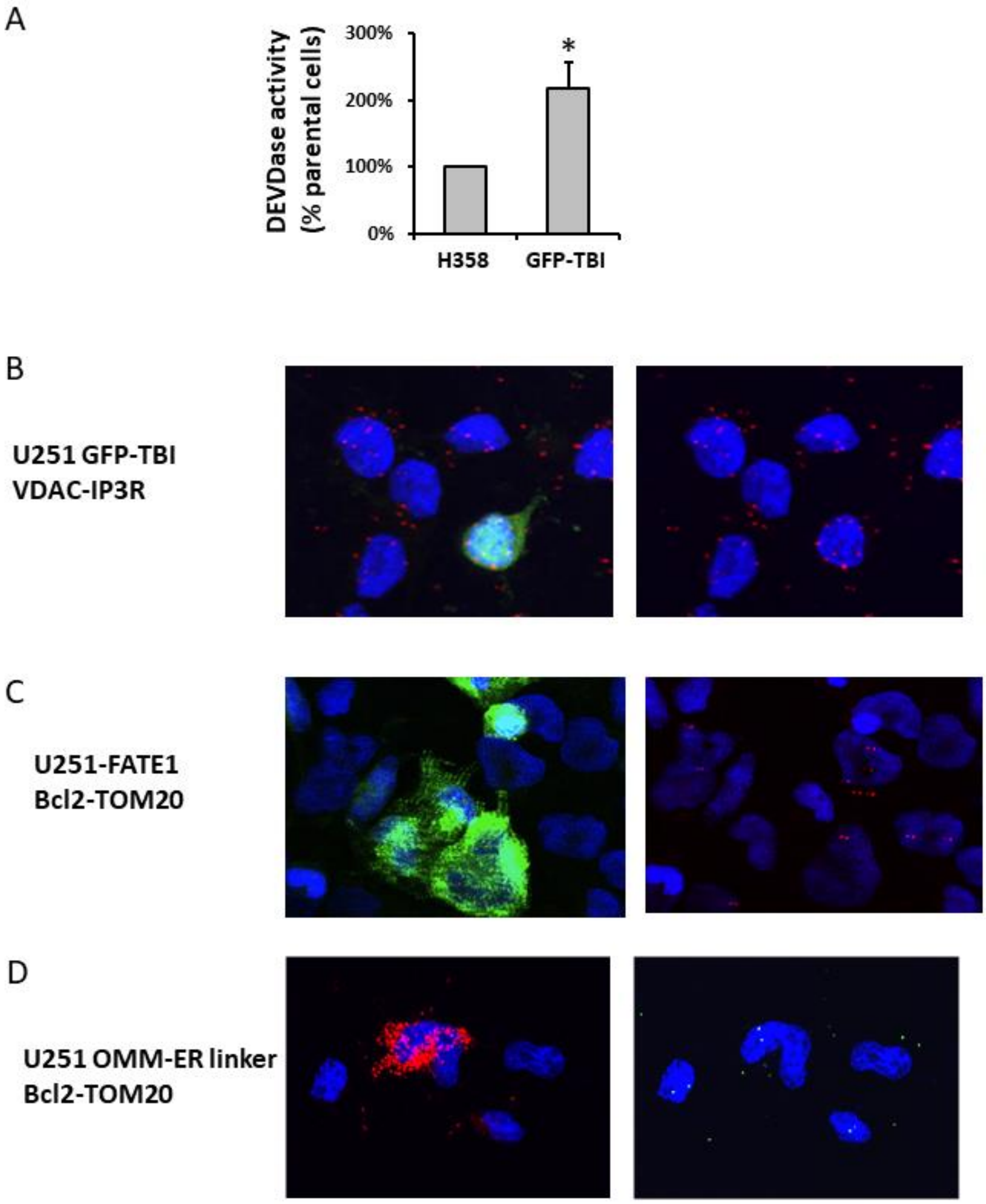
A
DEVDase activity
(% parental cells)
300%
200%
100%
0%
*
H358
GFP-TBI
B
U251 GFP-TBI
VDAC-IP3R
C
U251-FATE1
Bcl2-TOM20
D
U251 OMM-ER linker
Bcl2-TOM20

Figure S3

Figure S4: A) Yeast or human TOM20 expression was induced in S.cerevisieae. TOM20 expression was analysed by western blot in whole cell lysates and in isolated mitochondria. B) Human Bcl-xl was expressed in the cells described in A. Bcl-xl mitochondrial localization was analysed by mitochondria isolation and western blot. Mitochondrial Bcl-xl was quantified by the Bcl-xl/Porin ratio. C) Human Bcl2Maob or Bcl2cb5 were expressed in yeast and mitochondria were isolated. Bcl2 expression was measured by western blot in mitochondrial pellet and in the supernatant. GFP-TBI was then expressed and the localization of Bcl2 was measured as described above. GFP-TBI inhibits the mitochondrial localization of both Bcl2Maob and Bcl2cb5. D) Whole cell lysates were loaded on top of a 25-65% Optiprep gradient. After a 16-hours centrifugation at 105,000 x g, gradients were fractionated in 10 fractions that were analyzed by SDS-PAGE and western-blotting againt Pgk1 (cytosolic marker), Por1 (mitochondrial marker) and Bcl-2. Bcl-2 amounts in PMS fractions (containing Pgk1) and mitochondrial fractions (containing Por1) was reported on Figure 4D.

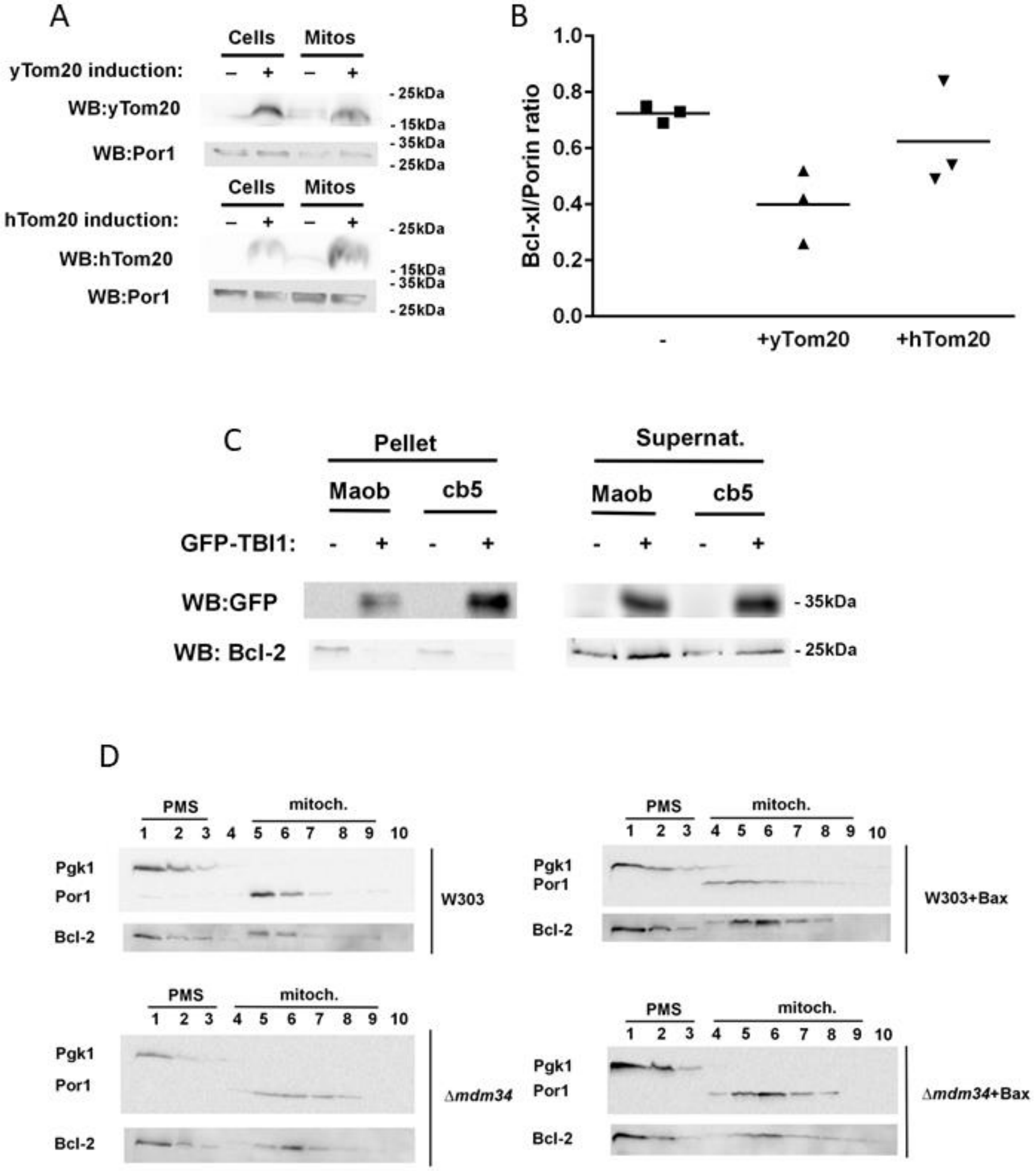
A
Cells
Mitos
yTom20 induction:
WB:yTom20
WB:Por1
hTom20 induction:
WB:hTom20
- 25kDa
- 15kDa
- 35kDa
B
Bcl-xl/Porin ratio
+yTom20
+hTom20
C
Pellet
Supernat.
Maob
cb5
GFP-TBI1:
WB:GFP
WB: Bcl-2
D
PMS
mitoch.
Pgk1
Por1
Bcl-2
W303
W303+Bax
Δmdm34
Δmdm34+Bax

Figure S4

Figure S5: U251 cells were treated by ABT737 for 24h to prevent Bcl2-Bax interaction, then STS was added in the indicated cells for the last 4h. Cells were fixed and Bcl2-Bax and Bcl2-TOM20 interactions were detected by PLA as described in the article. The graph represents the number of spots per cell in each condition (each point is an individual count) (***: $p<0.001$ in STS vs ABT737+STS).

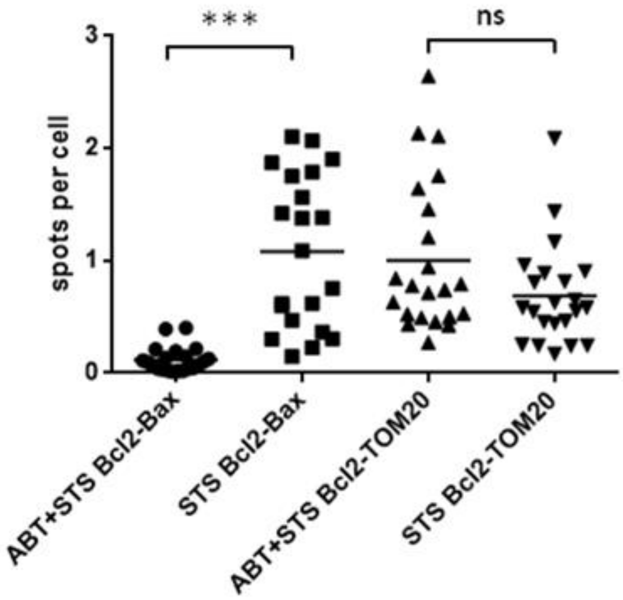
***
ns
3
2
1
0
spots per cell
ABT+STS Bcl2-Bax
STS Bcl2-Bax
ABT+STS Bcl2-TOM20
STS Bcl2-TOM20

Figure S5
